# Bipolar patients display stoichiometric imbalance of gene expression in post-mortem brain samples

**DOI:** 10.1038/s41380-023-02398-0

**Published:** 2024-02-13

**Authors:** Asbjørn Holmgren, Ibrahim Akkouh, Kevin Sean O’Connell, Jordi Requena Osete, Pål Marius Bjørnstad, Srdjan Djurovic, Timothy Hughes

**Affiliations:** 1 Department of Medical Genetics, Oslo University Hospital, Oslo, Norway; 2 Norwegian Centre for Mental Disorders Research, Division of Mental Health and Addiction, Oslo University Hospital & Institute of Clinical Medicine, University of Oslo, Oslo, Norway; 3 NORMENT, Department of Clinical Science, University of Bergen, Bergen, Norway

**Keywords:** Genetics, Bipolar disorder, Molecular biology

## Abstract

Bipolar disorder is a severe neuro-psychiatric condition where genome-wide association and sequencing studies have pointed to dysregulated gene expression as likely to be causal. We observed strong correlation in expression between GWAS-associated genes and hypothesised that healthy function depends on balance in the relative expression levels of the associated genes and that patients display stoichiometric imbalance. We developed a method for quantifying stoichiometric imbalance and used this to predict each sample’s diagnosis probability in four cortical brain RNAseq datasets. The percentage of phenotypic variance on the liability-scale explained by these probabilities ranged from 10.0 to 17.4% (AUC: 69.4–76.4%) which is a multiple of the classification performance achieved using absolute expression levels or GWAS-based polygenic risk scores. Most patients display stoichiometric imbalance in three to ten genes, suggesting that dysregulation of only a small fraction of associated genes can trigger the disorder, with the identity of these genes varying between individuals.

## Introduction

Bipolar disorder (BD) is a severe psychiatric illness characterised by episodes of depression and mood elevation with a prevalence of 1–2% and first onset typically in late adolescence to early adulthood [1]. This early onset causes detrimental effects for personal well-being over the lifespan and a considerable disease burden worldwide [2]. Twin studies have estimated the broad-sense heritability of BD to be over 60%, indicating that genetic factors play a dominant role in the aetiology of the disorder [3].

The latest genome-wide association study (GWAS) analysis encompassing 41,917 cases and 371,549 controls, found 64 genome-wide significant loci with the SNPs of each locus having a relatively small effect (odds ratio <1.15 for the risk allele) [4]. One way of summarising GWAS results is the computation of a polygenic risk score (PRS) for BD from an individual’s genotype data [5]. Using the latest published GWAS (PGC3), the percentage of phenotypic variance on the liability-scale explained by PRS is 4.57% and the weighted mean area under the ROC curve (AUC) is 65% [4]. Although this is an improvement from earlier GWAS [6], this predictive accuracy of BD is still modest and far from the 80% considered necessary for clinical utility [7, 8].

Most of the GWAS variants are located in non-coding intergenic regions, suggesting that they act through their effect on gene transcription, either directly (promoters and transcription factor binding sites) or indirectly (epigenetic marks and chromosome conformation). Further, a recent whole-exome sequencing study of coding variants in 13,933 cases and 14,422 controls, found that risk genes implicated through GWAS are not enriched for rare coding variants [9], thus lending further support to the hypothesis that dysregulation of transcription is the main cause of the disorder. On the other hand, other studies suggest that dysregulation of transcription plays only a minor role in disease aetiology [10]. Substantial effort has been invested in directly testing for case-control gene expression differences in human brain samples, and there are promising results at the gene-group level, with enrichment of certain biological functions. For instance, groups of genes with spatial and functional ties to the postsynaptic density have been reported as jointly differentially expressed in BD in several brain regions [11–13] and a co-expressed gene-module enriched for microglial-associated genes was shown to be significantly down-regulated in BD [13]. Attempts to combine genotypes with effects on gene expression have shown some interesting findings. For instance, expression quantitative trait loci (eQTL) evidence from sub-genual anterior cingulate cortex (sACC) have pointed to genes coding for ion channel subunits *SCN2A* and *GRIN2A* [13]. However, these studies which have primarily taken a transcriptome-wide approach and focused on absolute expression levels, have detected very little statistically significant case-control differential expression (DE) of genes identified by GWAS [11, 13, 14].

In this study, we first analysed four case-control cortical brain RNAseq datasets [11, 13, 15], focusing only on the GWAS-associated genes to determine whether there are any patterns in absolute DE that are consistent across datasets. Second, the previous observation of gene co-expression modules [11, 13] suggested that many genes may be under evolutionary pressure to maintain relative rather than absolute expression levels. We therefore hypothesised that such a stoichiometric constraint applies to many of the GWAS-associated genes i.e. that the ratios in expression between subsets of genes need to be kept within a certain range to ensure healthy brain function and that BD may be caused by stoichiometric imbalance (SI) rather than abnormal absolute expression levels. We developed two methods for quantifying the level of stoichiometric imbalance in an RNA sample and evaluated their diagnostic classification performance compared to the PRS. Finally, we dissected the architecture of the stoichiometric imbalance at the gene and individual levels to gain detailed insight into its role in BD aetiology.

## Methods

### Human brain expression data

RNA sequencing data of post-mortem brain dorsolateral prefrontal cortex were obtained from the collections CommonMind [15] and BrainGVEX [11]. In addition, data from subgenual anterior cingulate cortex (sACC) were obtained from BipSeq [13].  Samples from subjects below 18 years of age were excluded, as the case group did not include children and this case-control imbalance could potentially lead to a confounding of BD etiology with age-related brain development. [16]. Details on sample sizes, age, sex, post mortem interval (PMI), RNA integrity number (RIN), sequencing methods and mapped reads are given in Table 1.

**Table 1:** Case-control brain RNAseq datasets in BD.

Dataset	Region	Samples		Mean age		Cauc.	Male	PMI	RIN	Mapped reads
		BD	HC	BD	HC					
CMC-HBCC	DLPFC	71	165	42.6	43.1	50.0%	72.0%	28.0	7.7	103
CMC-Pitt	DLPFC	35	93	45.5	47.8	89.8%	66.1%	20.7	8.4	75.6
BrainGVEX SMRI	DLPFC	73	75	44.0	47.0	95.9%	66.2%	28.0	7.9	45.7
BipSeq sACC	sACC	125	142	42.5	50.5	100.0%	72.1%	27.0	7.7	110
*BipSeq Amygdala*	*Amygdala*	*120*	*122*	*42.9*	*52.3*	*100.0%*	*70.8%*	*27.0*	*7.3*	*106*
*BrainGVEX BSHRI*	*DLPFC*	*0*	*184*	*NA*	*74.6*	*99.5%*	*58.7%*	*2.7*	*6.9*	*50.5*

*DLPFC* dorsolateral prefrontal cortex, *sACC* subgenual anterior cingulate cortex, *PMI* Post mortem interval in median number of hours, *RIN* RNA integrity number, median across cohort samples.

All samples were prepared using rRNA depletion with stranded prep, with the exception of CMC-Pitt which was prepared unstranded. All samples were sequenced 100 bp paired-end.

BipSeq-Amygdala and BrainGVEX-BSHRI in italics as they were not used in this study (see methods).

Read counts mapped to genes for each dataset were between-sample normalised using the weighted trimmed mean of M-values (TMM) with edgeR [17]. Expression values were then within-sample normalised to transcript lengths and library size (RPKM). Gene-level transcript lengths were obtained from Ensembl version GRCh38.99 (Homo_sapiens.GRCh38.99.gtf), using the canonical transcript or the median length of transcripts where no canonical transcript was defined. Genes with low expression (<10 counts in >30% of the samples, converted to CPM for the median library size) were filtered out with the “filterByExpr” function in the edgeR package.

From the CommonMind, the cohorts NIMH Human Brain Collection Core (CMC-HBCC) (BD = 71, HC = 165) and CMC-Pitt (BD = 35, HC = 93) were included. From BipSeq, the sACC samples were included (BD = 125, HC = 142), but the amygdala samples were not as they are not cortical. These three sample sets have a similar age profile (Table 1). In BrainGVEX, the SMRI cohort was included (mean age = 47, BD = 73, HC = 75), but the BSHRI cohort was not because it consists exclusively of controls with a significantly older age profile (mean age = 75). We performed principal component analysis (PCA) in all datasets and visually inspected the first and second principal components. We detected two outlier samples in the CMC-HBCC which were removed from the subsequent analysis (Fig. S1).

### Gene set

The BD Working Group of the Psychiatric Genomics Consortium (PGC3) identified 64 genome-wide significant loci. We used the OpenTargets’ locus-to-gene measure (L2G) [18] to prioritise one gene from each locus. The L2G measure combines genetic distance, eQTL results, chromatin interaction and variant pathogenicity into a prediction score (0-1) [19]. For each of the 39 loci with at least one gene with L2G > 0.5, we used the protein-coding gene with highest L2G (Tables S1, 2). Two loci each had two genes with very similar L2G, and both of these genes were used. For the 21 loci with no gene achieving L2G > 0.5, we used the protein-coding gene closest to the GWAS index SNP (Table S3). There are four loci without any protein-coding genes, and there are two loci with overlapping gene sets. This results in 61 unique genes, of which 54–55 (depending on dataset) have sufficient expression data to be analysed in our datasets. We define this set of genes as the PGC3 GWAS genes.

### Residualised expression levels (absolute and relative)

For absolute expression levels, we regressed the normalised expression levels of each gene (log_2_ RPKM) against the five covariates (age, sex, ethnicity, RIN and PMI) and computed the residualised absolute expression levels.

For relative expression levels, the procedure was more complex. We modelled each gene’s expression (log_2_ RPKM) as a linear function of the other genes in the set as well as the known covariates age, sex, ethnicity, PMI, and RIN. Since there is a high degree of correlation in expression between many of the genes and because these models have a high number of variables relative to the number of samples, we fitted them by Least Absolute Shrinkage and Selection Operator (LASSO) penalised regression with the R package glmnet [20]. We used 10-fold cross-validation to find the optimal value of regularisation parameter λ that gives the most regularised model such that the cross-validated error is within one standard error of the minimum. We set the glmnet function’s ‘penalty.factor’ parameter for the sex, age, ethnicity, RIN and PMI variables to 0 to ensure these variables are always included unpenalised in the model.

**Fig. 1:**
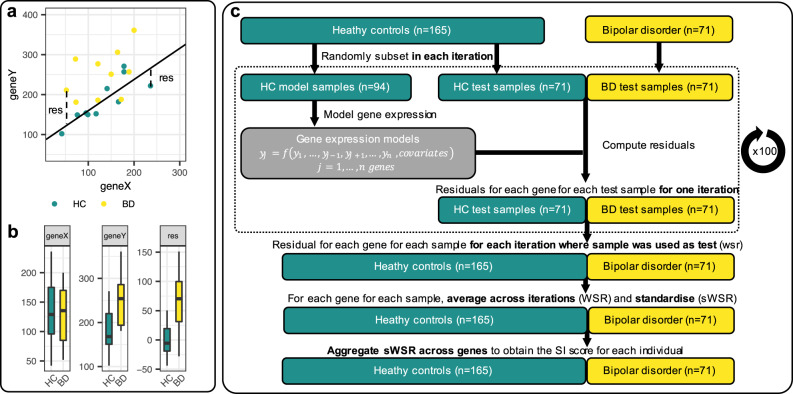
SI concept and SI metric calculation in CMC-HBCC. **a** Illustration of the SI concept in the simplified scenario of only two genes. Gene X and Y have correlated expression and the solid line depicts the linear regression fit to the HC samples. “res” = residuals from the regression line. Here, the residual is a measure of the extent to which a gene Y’s expression is in stoichiometric imbalance with gene X (with a residual of 0 indicating perfect stoichiometric balance). **b** HC and BD samples have a similar range of absolute expression levels for gene X. For gene Y, BD levels are higher than HC, but perhaps not significantly so. However, the residuals (of gene Y modelled as a function of X) are clearly higher in BD samples with no overlap in the boxes. **c** Illustration of the resampling strategy, exemplified with the CMC-HBCC dataset. The gene expression model cannot be fitted and tested on the same HC samples, as this would introduce a bias for lower residuals in the HC samples relative to BD. Instead, the HC samples are split into a set used for fitting the models and a test set for which residuals will be calculated. This ensures that when applying a gene model to compute a sample’s residual, the sample was not used in fitting the model. In order to avoid the final result being determined by one random sampling and in order to obtain residuals for all HC samples, we iteratively perform random sampling, fitting, and residual calculation. Residuals are then averaged across iterations and standardised, before being aggregated in the SI score.

Since we detected case-control differences in absolute expression levels of several genes, we cannot fit the models to samples irrespective of diagnosis. Instead, we limit the modelling to control samples. To not introduce a bias that systematically produced lower residuals in controls than in cases, these models were fitted using a control sample set that did not overlap with the test sample sets. For example, in the CMC-HBCC data set, we split the control set into 94 modelling samples and 71 test samples, then we fitted the gene models using the modelling samples, and finally we computed the residuals in the non-overlapping 71 control and 71 case test samples (Fig. 1c). If we had done the random sub-setting of controls into model and test samples only once, our results would have been dependent on that one random sub-setting. To avoid this, we resampled randomly 100 times, each time changing the random set of 94 control samples used to build the models, and keeping the rest of the controls (*n* = 71) as well as the cases (*n* = 71) to calculate the residuals. There were never any overlapping samples between the modelling and the test set, thus limiting the possibility of any bias that would give controls a better fit to the model than BD cases.

Datasets differ in total size and case-control composition which created the need for different resampling strategies to ensure sufficient data for modelling and sufficient testing of all controls. Model/test split of control samples and number of iterations in parenthesis were: CMC-HBCC 94/71 (100), CMC-Pitt 69/23 (100), BrainGVEX-SMRI 67/8 (1000), BipSeq-sACC 93/47 (100).

We defined the scaled residuals as the difference between each observed and predicted gene expression value divided by the mean observed value for this gene across all samples. The fits of the expression models differed somewhat between iterations for the same gene and differed highly between genes (Fig. S2), we therefore also weighted the scaled residuals (WSR) by the *R*^2^ of the gene model from each iteration (${{wsr}}_{i,j}^{k}$, Eq. (1)). The gene model is not based on any of the case samples or any of the control samples that were chosen to be in the test set in that iteration. We averaged the ${{wsr}}_{i,j}^{k}$ across iterations (in which individual i was not used in modelling) to obtain one value for each gene for each sample (${{WSR}}_{i,j}$, Eq. (2)).

- $i=1,\ldots ,m$ individuals in the full dataset
- $$j=1,\ldots ,n\,{genes}$$
- For $k=1,\ldots ,p$ iterations, choose a subset of controls ${M}^{k}$ as the modelling set.
- Fit $n$ gene models ${y}_{j}=f\left({y}_{1},\ldots ,{y}_{j-1},{y}_{j+1},{\ldots ,y}_{n},{covariates}\right)$ using individuals in ${M}^{k}$.
- $${{R}^{2}}_{j}^{k}={R}^{2}\,{of}\,{modelling}\,{gene}\,j\,{in}\,{iteration}\,k$$
- $${\hat{y}}_{i,j}^{k}={predicted}\,{expression}\,\left({\log }_{2}{RPKM}\right)\,{of}\,{gene}\,j\,{for}\,{individual}\,i\,{in}\,{iteration}\,k$$

1 $${{wsr}}_{i,j}^{k}={{R}^{2}}_{j}^{k}\frac{{y}_{i,j}-{\hat{y}}_{i,j}^{k}}{\frac{1}{m}\mathop{\sum }\nolimits_{l=1}^{m}{y}_{l,j}\,}$$

2 $${WS}{R}_{i,j}=\frac{1}{{number}\,{of}\,{iterations}\,{where}\,i\notin {M}^{k}}\mathop{\sum}\limits_{k:i\notin {M}^{k}}{ws}{r}_{i,j}^{k}$$

To make the residualised absolute and relative expression values comparable across genes and across datasets, we standardised all samples (cases and controls) by subtracting the HC mean and dividing by the HC standard deviation (within each dataset). For the relative expression levels, we denote these standardised ${{WSR}}_{i,j}$ as ${{sWSR}}_{i,j}$.

### Tests of differential gene expression (absolute and relative)

At the gene level within datasets, we tested for differences in residualised absolute expression and residualised relative expression (${{WSR}}_{i,j}$) between cases and controls using regular Wilcoxon test, adjusting for multiple testing across all genes and datasets using the Benjamini-Hochberg method with FDR of 0.05 [21]. Tests could also have been performed on the ${sWSRs}$ and would have yielded the same results as the standardisation is a linear transformation.

For the absolute expression levels, we also performed a second, more powerful test that took the unresidualised expression levels as input. The TMM-normalised counts from edgeR’s DGEList object were analysed for differentially expressed genes using the limma (3.46.0) package in R with voom transformation (10.1186/gb-2014-15-2-r29), including the known covariates age, sex, PMI, and RIN. Linear models were fitted with the lmFit function and empirical Bayes were used to obtain more precise estimates of gene-wise variability [22]. The PGC3 genes were extracted from the result, and nominal p-values of the DE test were adjusted with the Benjamini-Hochberg method with FDR of 0.05.

### The SI score

For the relative expression levels, we define the individual stoichiometric imbalance (${{SI}}_{i}$ Eq. (3)) as the mean absolute value of ${{sWSR}}_{i,j}$ across all genes for that individual.

3 $${{SI}}_{i}=\frac{1}{n}\mathop{\sum }\limits_{j=1}^{n}\left|{{sWSR}}_{i,j}\right|$$

### Predicting diagnosis with cross-validated logistic regression

For both the relative and the absolute expression, we estimated diagnosis probability using the standardised-residualised expression levels. For each cortical dataset, we fitted a logistic regression of diagnosis against the standardised-residualised expression of every gene (relative or absolute), using the samples of the three other cortical datasets. We used the fitted model (Table S4) and the standardised-residualised expression (relative or absolute) of the dataset of interest to obtain the predicted probabilities of diagnosis.

### Diagnostic performance tests

We evaluated the SI score’s and predicted probability’s ability to discriminate between cases and controls in three different ways: 1. we tested for statistically significant differences in the metric between cases and controls using Wilcoxon tests, 2. we computed the area under the receiver operator characteristic curve (AUC), and 3. we performed a logistic regression of diagnosis as a function of the metric and computed the Nagelkerke pseudo *R*^2^ which we adjusted to the liability scale to account for the higher proportion of cases in the sample set compared to the general population (using a BD population prevalence of 2% [4]).

### Control analyses

We performed two control experiments to empirically verify that our procedure for modelling relative expression levels in controls does not produce a bias that may inflate residuals in cases relative to controls. In the first, we switched to using a subset of the cases in each modelling iteration and then proceeded in the same way, using the mean and sd of the cases for scaling (Table S7).

In the second, using the two datasets with sufficiently many control samples (CMC-HBCC and BipSeq-sACC), we split the controls into two groups, and used a subset of the first group for modelling (with random resampling) and used the second group for testing (Table S6).

### Polygenic risk scores

The PRS scores for the PGC3 GWAS cohorts were obtained from the PGC3 study [4]. For the samples in the expression datasets, the PGC3 GWAS summary statistics were pruned for LD using the p-value-informed clumping method in PLINK v1.90 (*R*^2^ 0.1 within a 500-kb window) based on the LD structure of the HRC reference panel. Subsets of SNPs were selected from the results below nine increasingly liberal *p* value thresholds (GWAS PT; 5 × 10^−8^, 1 × 10^−4^, 1 × 10^−3^, 0.01, 0.05, 0.1, 0.2, 0.5, 1). Sets of alleles, weighted by their log odds ratios from the GWAS, were summed into PRSs for each individual in the target datasets, using PLINK v1.90. Genotype information was available for CMC-HBCC, CMC-Pitt, and BrainGVEX-SMRI. For the 290 individuals with expression data in BipSeq, we were only able to obtain genotypes for 172, so this cohort was excluded from this analysis. The cohorts used for PRS calculations in the PGC3 BD GWAS are a mix of European ancestry from multiple European countries and the US. We therefore also included an analysis where only samples with Caucasian ethnicity were kept.

## Results

### Absolute levels—differential gene expression and correlation analysis

For each of the 64 genome-wide significant loci [4], we identified the protein-coding gene that is most likely to be the source of the association (‘PGC3 GWAS gene’). We identified 61 unique protein-coding genes, of which 54–55 (depending on dataset) had sufficient expression data to be analysed (see methods). In all four case-control cortical datasets (Table 1), feature counts were normalised, converted to log_2_RPKM values, and residualised for age, sex, ethnicity, RNA integrity number (RIN), and post-mortem interval (PMI) (see methods). In a PCA of the gene expression values we observe that the DLPFC datasets group together and the sACC is separate on the first principal component (PC1) (Fig. S3a). A PCA of the residualised expression measures (Fig. S3b) shows that there is no clustering of datasets.

**Fig. 2:**
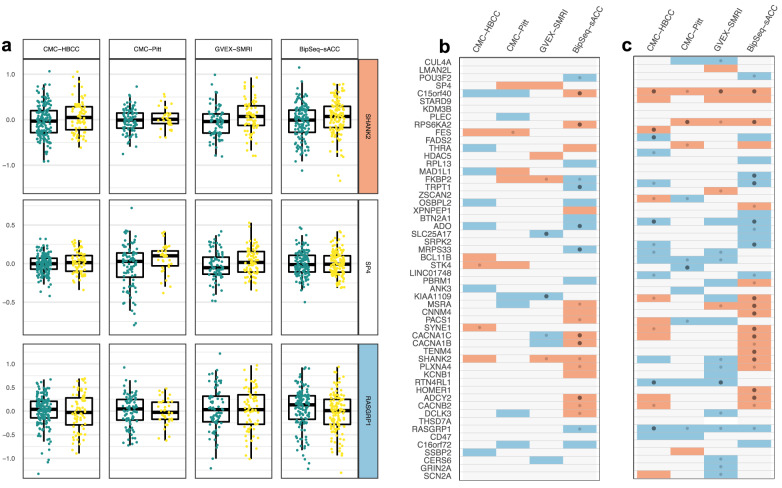
Patterns of case-control differences in absolute gene expression across datasets. **a** HC (green) and BD (yellow) gene expression, log2 RPKM residualised for covariates, for three example genes in all four data sets (nine outlier points are not shown in the plot). For *SHANK2* (top) cases have higher observed expression than controls. For *SP4* (middle), there is no pattern of difference between controls and cases, and for *RASGRP1* (bottom) cases have lower observed expression than controls. **b** *P* values from the comparison between HC and BD mean residualised expression for all PGC3 associated genes for all four datasets (gene ordering identical to Fig. 3). Colours indicate direction of difference; red higher in BD and blue lower in BD (*p* value < 0.15). Small dots indicate nominal significance (*p* < 0.05) and large dots indicate significant *p* value (FDR < 0.05 across all genes and datasets). **c** same as (**b**)., but for the LIMMA-voom differential gene expression statistics.

We tested each gene for DE with a simple Wilcoxon test (Fig. 2a, b) and with the more powerful limma-voom package which takes the unresidualised expression as input and controls for covariates internally (Fig. 2c) (see methods). In the BipSeq-sACC dataset, we found 24 genes that were nominally differentially expressed, with 15 of these surviving correction for multiple testing. Several of these differentially expressed genes in BipSeq-sACC also showed nominally significant DE in the same direction in at least one of the other three datasets (Fig. 2c).

**Fig. 3:**
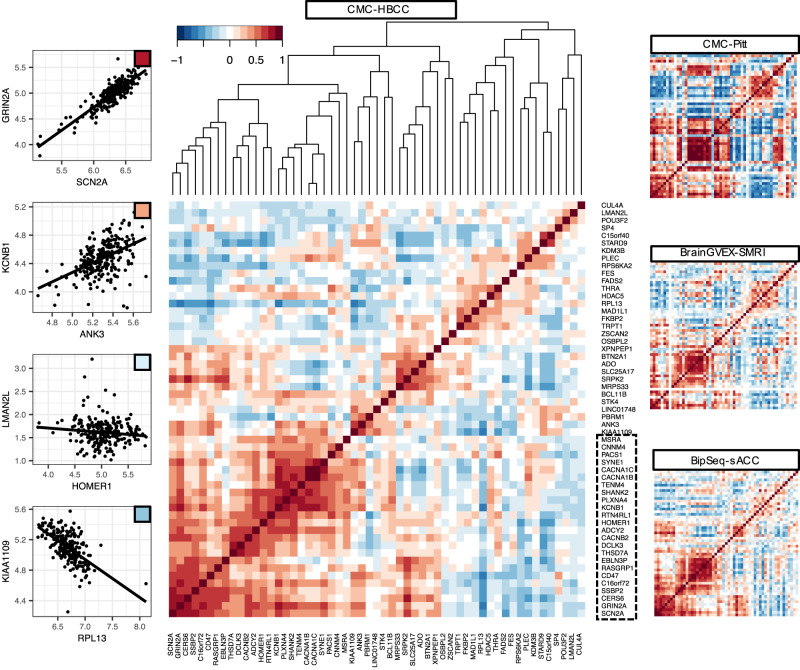
Expression correlation in four brain datasets for GWAS-associated genes. Left column: Four examples of gene-gene absolute expression correlation (Pearson) with the correlation level indicated with colour scale. Centre: The correlation between all pairs of PGC3-associated genes for the CMC-HBCC set, with genes ordered according to the hierarchal clustering displayed above the figure. Dotted line delineates genes in large module of co-expression. Right: Equivalent gene-gene expression correlations in the three other datasets, with the genes in the same order as for CMC-HBCC.

For each of the 54 GWAS genes, we computed the Pearson correlation of that gene’s normalised expression with that of the gene from each of the other loci. We performed the analysis in the four case-control datasets (Table 1) and observed strong patterns of co-expression between several subgroups of genes which replicated in all datasets (Fig. 3). In particular, we observed a large module of 24 genes with strong positive co-expression, as well as several smaller such modules which were often negatively correlated with each other.

### Relative levels—modelling and case-control differences

The clusters of co-expressed genes suggest that relative levels of expression are under tight regulatory control. We hypothesised that the normal function of a significant fraction of these genes may be dependent on the balance of their relative expression levels and that BD is the result of imbalance. When this imbalance of relative gene expression is characterised in a sufficiently large cohort, it is detectable as differential expression of absolute expression levels, as observed in BipSeq-sACC. If our hypothesis is correct, it should be possible to accurately predict the expression level of a gene in a healthy control (HC) individual from that individual’s expression levels of the other PGC3 genes (Fig. 1a) and BD patients will display a weaker fit to the model than controls (larger residuals), indicating stoichiometric imbalance (Fig. 1b).

To predict the expected expression level of each gene, we used the LASSO method to fit a model of that gene’s expression as a function of the expression of the other genes and known covariates. Since we detected case-control differences in absolute expression levels of several genes, we cannot fit the models to samples irrespective of diagnosis. Instead, we limit the modelling to control samples which are randomly resampled (Fig. 1c, see methods). We found that many genes have well-fitting models with *R*^2^ above 0.75 and that there is strong consistency in the best fitting models across datasets (Fig. S2). Unsurprisingly, many of the best models are for genes in the highly co-expressed module (Fig. 3), but many genes outside the module also have good fit. For each individual, we computed the predicted expression level for each gene (given the model and the observed explanatory variables) and the residual (difference between observed and predicted level). The size of residuals varied greatly between genes, increasing with the gene’s normalised expression level and decreasing with gene model fit. We defined the weighted scaled residual for individual *i* and gene *j* in resampling iteration *k* (${{wsr}}_{i,j}^{k}$) as the residual from iteration *k* multiplied by the R^2^ of gene *j*’s model in iteration *k* and divided by the observed expression of gene *j* (averaged across all individuals), and ${{WSR}}_{i,j}$ as the mean of ${{wsr}}_{i,j}^{k}$ across iterations (see methods, Eqs. (1) and (2)).

**Fig. 4:**
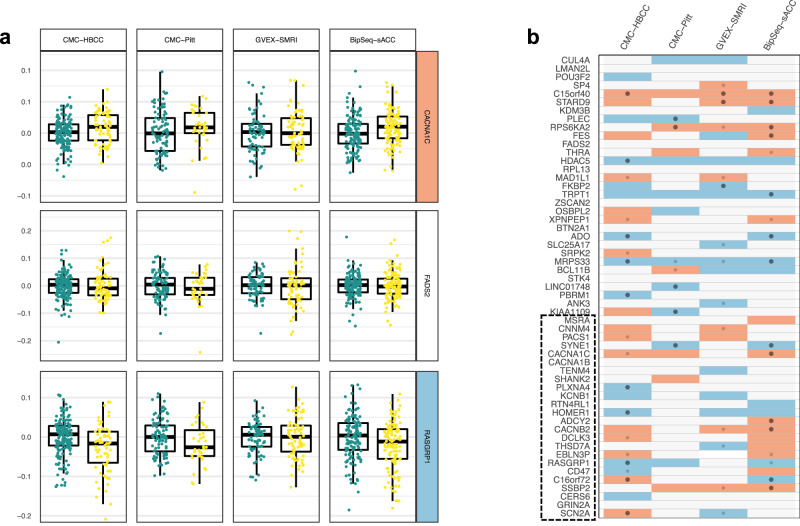
Patterns of case-control differences in relative gene expression across datasets. **a** HC (green) and BD (yellow) residuals (${{WSR}}_{i,j}$ weighted scaled residuals averaged across iterations) for three example genes in all four datasets (5 outlier points are not shown in the plot). For *CACNA1C* (top) cases have higher observed expression than predicted. For *FADS2* (middle), there is no pattern of difference between controls and cases, and for RASGRP1 (bottom) cases have lower observed expression than predicted. **b** *P* values from the comparison between HC and BD mean residuals for all PGC3 associated genes for all four datasets (gene ordering identical to Fig. 3, dotted line delineates boundary of large module of co-expression). Colours indicate direction of difference; red higher in BD and blue lower in BD (p-value < 0.15). Small dots indicate nominal significance (*p* < 0.05) and large dots indicate significant p-value (FDR < 0.05 across all genes and datasets).

Many genes did not display ${{WSR}}_{i,j}$ case-control differences (Fig. 4a) in any datasets (e.g. *FADS2*), but many others displayed either a relative over-expression in cases (e.g. *CACNA1C*) or relative under-expression (e.g. *RASGRP1*). We performed Wilcoxon statistical tests of case-control mean differences in ${{WSR}}_{i,j}$ for all genes in all datasets (Fig. 4b). We found that there was generally good consistency in this direction of effect across datasets and that many differences were statistically significant even after correction for multiple testing across all genes and datasets, with CMC-HBCC (10 genes) and BipSeq-sACC (13 genes) standing out in this respect. Further, there was a matching direction of effect between the absolute (Fig. 2b, c) and relative DE analyses (Fig. 4b). For the 23 genes with FDR-corrected significance in at least one dataset (Fig. 4b), there was only one gene (*C16orf72*) with inconsistent direction of effect across datasets that was FDR-corrected significant, and one further example (*SCN2A*) of inconsistency in direction of effect when the criterium is loosened to nominally significant differences. Additionally, there was a roughly equal number of genes affected by over-expression and by under-expression (Figs. 2c and 4b) and no obvious correlation between genes with better models (Fig. S2) and significant case-control differences in ${{WSR}}_{i,j}$.

### Relative levels—stoichiometric imbalance score

**Fig. 5:**
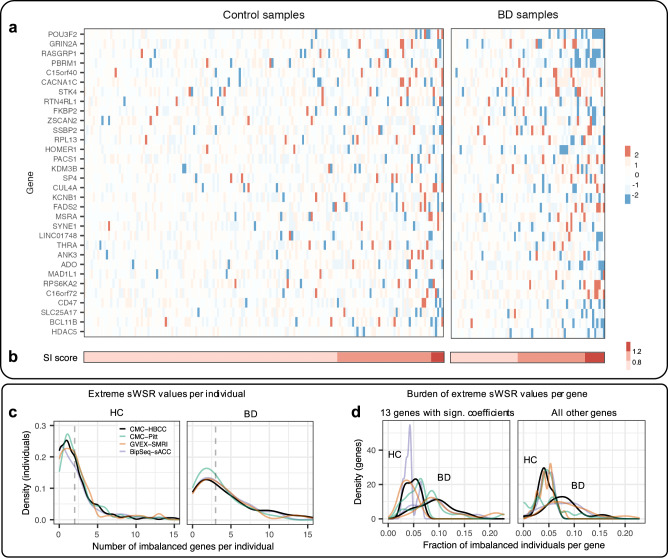
Stoichiometric imbalance across genes and individuals. **a** and **b**: SI score computation in CMC-HBCC. **a** Standardised, weighted scaled residuals averaged across iterations (${{sWSR}}_{i,j}$). Rows are genes sorted according to mean residuals in the BD set (with most deviant genes on top). Only the top 32 deviant genes are shown. Columns are samples sorted with increasing SI score. **b** Aggregation of each individual’s residuals across genes according to the SI score definition (Eq. (3)). **c** and **d**: Polygenic nature of BD aetiology with imbalanced defined as $\left|{{{sWSR}}}_{{{i}}{l{,}}{{j}}}\right|{{ > }}{{2}}$. **c** Left: distribution of the number of imbalanced genes per healthy control. Median of CMC-HBCC density marked by vertical dotted line. Right: identical figure for BD cases. **d** Left: distribution of the fraction of imbalanced individuals (cases or controls) per gene for the 13 genes with significant coefficients in the logistic regression fitted with all four cortical datasets (Table S9). Right: identical figure for all genes ranked outside top 13.

**Fig. 6:**
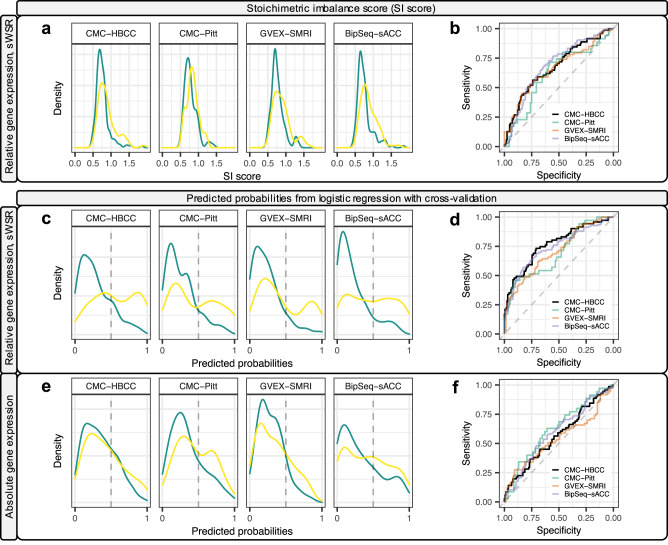
SI score and predicted probabilities. **a** Density distribution of SI score in each dataset (HC in green and BD in yellow) and **b** the associated ROC curves. **c** Density of the predicted probabilities estimated from the logistic regression of diagnosis as a function of relative gene expression (sWSR) and **d** the associated ROC curves. **e** Density of the predicted probabilities estimated from the logistic regression of diagnosis as a function of absolute gene expression and **f** the associated ROC curves.

We aimed to aggregate the WSRs across genes to obtain an SI score for each individual that can be used to classify samples. The weighting and scaling of residuals had resulted in values (${{WSR}}_{i,j}$) broadly in the same range (Fig. 4a), but there were still differences in the variance of HC values both between genes and between datasets. To further harmonise these values, we standardised case and control values by subtracting the HC mean residual and dividing by the HC standard deviation (s${{WSR}}_{i,j}$). A PCA analysis of the sWSR is plotted in Fig. S3c and shows that there is no clustering of datasets. We then aggregated the residuals across all 54 PGC3 genes by computing the mean absolute value of s${{WSR}}_{i,j}$ to obtain the individual’s SI score (Eq. (3), Fig. 5a, b) and plotted their distribution (Fig. 6a). We computed three tests of diagnostic performance: a Wilcoxon test for mean difference between cases and controls, the AUC (Fig. 6b), and we fitted the logistic regression of BD diagnosis against the SI score to obtain the Nagelkerke pseudo-R^2^ which was adjusted to the liability scale (assuming BD population prevalence of 2% [23]). For the three largest datasets (CMC-HBCC, BrainGVEX-SMRI, and BipSeq-sACC), this resulted in case-control differences significant at p = 7.8E-04 or better, AUC values greater than 66% and a liability-adjusted *R*^2^ of 4.8% or better (Table 2).

**Table 2:** Classification performance of metrics based on relative or absolute gene expression levels.

	Relative expression (sWSR)								Absolute expression			
	SI score				Predicted probabilities				Predicted probabilities			
Dataset	Genes	p	AUC	R^2^	Genes	p	AUC	R^2^	Genes	p	AUC	R^2^
CMC-HBCC	55	2.5E-05	0.67	5.3%	54	1.2E-10	0.76	17.4%	54	9.9E-02	0.57	1.3%
CMC-Pitt	55	3.7E-02	0.62	1.8%	54	7.4E-04	0.69	10.0%	54	2.8E-02	0.63	3.2%
BrainGVEX-SMRI	54	7.8E-04	0.66	6.1%	54	1.7E-05	0.71	10.2%	54	4.0E-01	0.54	0.9%
BipSeq-sACC	55	1.4E-07	0.69	4.8%	54	3.4E-11	0.74	13.5%	54	7.4E-03	0.60	1.8%

Predicted probabilities are estimated with validation across datasets: to predict the probabilities of a test dataset, the logistic regression model is fitted using the three other datasets and predicted probabilities are computed for the test dataset from the fitted model. This required removal of one gene that does not have expression in all datasets.

*p:* *p* value from the Wilcoxon Rank Sum test (Mann-Whitney-Wilcoxon) of difference between cases and controls.

*AUC:* Area under the ROC curve.

*R*^2^: Nagelkerke pseudo-R2 (liability-adjusted) from the logistic regression of diagnosis as a function of the SI score or the predicted probability.

### Logistic regression with cross-validation

Although the SI score displays highly significant case-control differences, it has a number of shortcomings. First, it includes all genes irrespective of whether they are informative in classifying samples, thus potentially adding noise. Second, it does not allow for effect sizes to differ between genes. We overcame these limitations by fitting a logistic regression of diagnosis against the standardised weighted scaled residuals (sWSR) of all 54 genes and perform cross-validation across cortical datasets (see methods and Table S4): we iteratively pick one dataset to be tested, fit the logistic regression model using the sWSR values of samples from the other three datasets, and then compute the predicted probabilities in the test dataset using the fitted model and the sWSR values of that dataset. The distribution of the predicted probabilities displays clear case-control differences (Fig. 6c) with a defined peak in the HC distribution close to zero and a bimodal distribution for BD cases in three of the datasets. This raised classification performance in all datasets with all AUC values in excess of 69% (Fig. 6d) and all liability-adjusted R^2^ above 10% (Table 2). The best performance was achieved in CMC-HBCC (AUC = 76.4%, *R*^2^ = 17.4%).

To compare the performance of the relative expression levels to what can be achieved with absolute expression levels, we performed the logistic regression of diagnosis as a function of the residualised absolute expression of all genes (with cross-validation). The absolute expression values were standardised using the HC mean and standard deviation in an identical fashion to the standardisation of the WSRs. The predicted probabilities were only marginally higher in cases (Fig. 6e) and the performance metrics were markedly worse than those derived from the sWSR (Fig. 6f) and were also mostly inferior to the performance of the SI measures (Table 2).

### Comparison to the polygenic risk score

The predicted probabilities computed from the relative expression levels have AUCs in excess of 69% in all datasets. However, it is possible that this high performance may be due to these datasets being biased towards BD patients with very high genetic risk. To test this, we computed the PRS for the three expression datasets where genotype data is available (see methods) and found that its AUC ranges between 28.5–54.2%, and 46.4–58.7% when only including samples of European descent (Table S5), which indicates that such a bias is not present.

For further context and a more challenging benchmark, we collated data on the performance of the PRS in the PGC3 BD GWAS sample. We computed the weighted mean metrics across case-control cohorts (35,421 cases and 55,774 controls, Table S6), using only genome-wide significant SNPs (97 SNPs) and using all SNPs significant at 10% (153,445 SNPs) and obtained liability-adjusted *R*^2^ of 0.7% and 4.6%, respectively. Thus, our predicted probabilities of BD (based on 54 relative gene expressions from genome-wide significant loci) outperform the most relevant comparator by more than an order of magnitude (CMC-HBCC 17.4%, CMC-Pitt 10.0%, BrainGVEX 10.2%, BipSeq-sACC 13.5%, Table 2) and also perform several times better than the 153k SNP PRS.

### Control analyses

Our gene expression modelling process, which uses a subset of the control samples (repeated with random re-samplings), was designed to avoid any systematic bias that would inflate the residuals in cases relative to controls. To verify this empirically, we performed control analyses in which we switched to performing modelling with case samples instead of controls. If there were a bias, we would then expect to see the control samples displaying SI. Instead, we observed that cases still had higher SI scores than controls with statistical significance comparable to modelling with controls (Table S7) and the patterns of over- and under-expression at the gene level were highly similar (Fig. S4). We also performed a second control analysis to check that our method returns a negative result when no SI is present. In the two datasets with large numbers of controls (CMC-HBCC and BipSeq-sACC), we split the controls into two groups and performed modelling with the first group. There were no statistically significant differences between the two groups, neither at the SI level (Table S8), nor at the gene level after correction for multiple-testing (Fig. S5).

### The polygenic nature of BD aetiology

It is unclear to what extent BD is characterised by each patient suffering from the dysregulation of a few genes that vary across patients, or by most associated genes being dysregulated in most patients. The ${{sWSR}}_{i,j}$ measures the deviation of the ${{WSR}}_{i,j}$ from the HC mean in units of HC standard deviation and can be used to define values that lie in the outer edges of the HC distribution (imbalanced value: $\left|{{sWSR}}_{i,j}\right| > 2$). We summarised each dataset’s gene-individual matrix of values (Fig. 5a) along both the individual and the gene dimension to gain insight into the disorder’s polygenicity. For controls, the median individual had approximately one to two imbalanced genes and very few individuals had more than five, whereas for cases, the median was three imbalanced genes and many individuals had more than five (Fig. 5c). Interestingly, even for cases, few individuals had more than 15 imbalanced genes. This suggest that dysregulation of a large number of the associated genes is not necessary to trigger the disorder, but disruption of only one or two tends to not be sufficient. To determine whether some genes are more frequently imbalanced than others, we compute the proportion of individuals (HC or BD in a dataset) in which a specific gene has an extreme value. The mean proportion was approximately 4.5% in HC, but was consistently higher in BD across all datasets (Fig. 5d). This pattern was strongest for the 13 genes with a significant effect on the predicted probability (*p* < 5%), as determined by a logistic regression of diagnosis against sWSR of all genes fitted using all samples from all datasets (Table S9). Interestingly, the pattern was also visible in genes with a less significant effect on the predicted probability. Further, the largest fraction of imbalanced individuals for a gene is 23% (Fig. 5d and S6), suggesting that there are no genes that are ubiquitously disrupted in BD. Instead, the data indicated high variance in the set of disrupted genes across individuals.

## Discussion

We focused on the expression levels of GWAS-associated genes and found consistent patterns of DE for many of these genes in four large case-control cortical brain RNAseq datasets. We also observed modules of strong co-expression between GWAS-associated genes which replicated across the four datasets. This is not unexpected given that many neurobiological processes are dependent on a delicate cellular balance of specific molecules for optimal function. For example, the electrophysiological properties of neurons are sensitive to the relative balance of ion channels and pumps [24]. This led us to hypothesise that SI may be part of BD aetiology. In four independent datasets, we found that many of the genes consistently displayed either relative under- or over-expression in BD patients and that this stoichiometric imbalance could be aggregated across genes to provide diagnostic classification at a level approaching clinical utility.

### Differential absolute expression of GWAS-associated genes

Several rounds of increasingly well-powered GWAS have been performed in BD, culminating in the PGC3 GWAS which identified 64 genome-wide significant loci (*p* < 5.0E-08). Transcriptome-wide searches for case-control differentially expressed genes in human brains have been performed in at least four case-control datasets [11, 13]. Typically, these studies have identified many hundreds of differentially expressed genes, but only a few were located in regions identified by GWAS. However, the transcriptome-wide approach potentially bears a high risk of failing to identify true DE in the GWAS loci. Indeed, the odds ratio (OR) of the lead SNPs in GWAS are small which suggests that any DE is likely to involve small fold changes that will have relatively low nominal significance in these RNAseq datasets of limited sample size. A transcriptome-wide search requires correcting for over 15k tests which is likely to lead to true DE being non-significant. A statistically valid alternative approach is to limit the test of DE to genes located in the 64 genome-wide significant loci and correct for multiple testing of this set of genes. This approach resulted in the identification of 15 genes that were FDR-corrected DE in the BipSeq-sACC dataset. Further, many of these genes displayed a consistent direction of effect in the other datasets and were also often nominally or FDR-corrected significant.

### Stoichiometric imbalance

The high correlation in expression level between GWAS genes, which replicated across datasets, led us to propose the SI hypothesis of BD. At the centre of our approach to testing this hypothesis, are models which predict each gene’s expression given the expression of other associated genes. Because of the case-control DE in absolute gene expression levels, the modelling was initially limited to controls. However, when modelling with the case samples, we obtained remarkably similar results (Table S7) even at the gene level (Fig. S4), thus empirically confirming that the results are independent of which samples are used to fit the models. This may seem counter-intuitive, but it is important to note that a gene’s expression model uses expression of all other genes as input variables and that each BD case is only subject to over- or under-expression in a small subset of genes, thus limiting biases in model fitting when using BD cases.

Although we identified 24 genes with FDR-corrected significant differential relative expression in at least one dataset, the remaining genes do not display significant case-control differences. For some loci, our methods may have failed to identify the correct gene or our models may not be accurate enough to capture the effect. Another important consideration is that we do not expect every locus to be associated with the disorder through a stoichiometric imbalance effect: some locus associations may be driven by genes that are only expressed early in neurodevelopment, or the basis of the association may be entirely unrelated to transcription. However, there are a sufficient number of genes that display differential relative expression and the direction of effect is sufficiently consistent across datasets that the cross-validated predicted probabilities reach as high as 76.4% in CMC-HBCC, which is close to the clinical utility threshold of 80%. Further, measured in liability-corrected *R*^2^, these predicted probabilities achieve a classification performance that is several times higher than what is achieved by the PRS in the GWAS cohorts (based on all 153k SNPs significant at 10%).

### Epistatic effects

The predicted probability suffers from two handicaps relative to the 153k SNP PRS. First, it incorporates expression from only 54 genes in the genome-wide significant loci (some of which may be misidentified). And second, expression, unlike genotype, may fluctuate over time such that the relative expression levels may not always be disrupted. Indeed, in cases, we observe a bimodal distribution of predicted probabilities (Fig. 6c) with one peak below 0.5 and one above. We suggest that the first peak may consist of patients in a euthymic state who were in stoichiometric balance at the time of death and are indistinguishable from controls.

The scale of the outperformance despite these handicaps suggests that our SI metrics capture an important aspect of disease aetiology which the PRS does not. GWAS and the PRS are both based on an assumption of independent additive effects between SNPs, whilst SI implies epistatic effects. Indeed, if the protein product of a gene is required to be in stoichiometric balance with another set of proteins, then the effect of a regulatory SNP would be dependent on the SNPs regulating stoichiometrically-related proteins. For example, the effect size of a SNP allele causing high transcriptional levels of a gene would depend on whether variation at other loci drives stoichiometric balance at a high or a low transcription level.

The results produced by GWAS continue to suffer from the missing heritability paradox whereby the proportion of phenotypic variance explained by the PRS is very low [4]. The three commonly proposed explanations for this deficit are rare coding variants, BD being highly polygenic with very small effect sizes that are difficult to quantify, and epistatic effects between variants [25]. In model organisms, systematic screens of genetic interactions affecting quantitative traits have shown the ubiquity of epistasis [26]. In humans, there is mounting general evidence that eQTLs identified by GWAS are context-dependent and non-additive [27], and there is also some direct evidence of synergistic effects between common risk variants, for example in schizophrenia [28]. However, statistical challenges in identifying epistatic interactions have made it difficult to quantify their contribution to the missing heritability [29, 30]. Our finding that a metric aggregating SI between genes explains a significantly higher fraction of the phenotypic variation than the PRS, suggests that non-additive epistatic interactions may be relevant to disease aetiology and may explain part of the missing heritability in BD. Further, SI may be relevant to other polygenic pathologies of the brain with large missing heritability, schizophrenia being an obvious candidate due to its high genetic correlation with BD [31].

### Limitations

We limited this study to genes with the strongest statistical association with BD i.e. those located in the genome-wide significant loci. As a result, genes that may be important for disease aetiology, but are located outside these loci, will have been excluded. And, even with this stringent approach, we cannot be certain that the gene identified for each locus is the one driving the GWAS association signal. Future work should aim to improve fine mapping as each correctly mapped locus adds to the number of informative genes, potentially improves the expression models of other genes, and thus may further improve the classification performance. A second limitation is that we have only investigated SI in bulk RNAseq from cortical brain regions. So, we are unable to determine whether the observed SI extends to other regions such as the temporal lobes, insula or corpus callosum which have all been implicated in BD brain imaging studies [32, 33]. A third important caveat is that gene expression may be affected by the cause of death and, in patients, may also be affected by drug treatments [34, 35]. Unfortunately, this data was either absent or incomplete in the available datasets.

Although the performance of the predicted probabilities approaches the clinical utility level of 80%, it is not clinically useful as it relies on measures of gene expression in inaccessible brain tissue. Future work should, therefore, aim to develop methods that can infer SI risk from genotype data which has the added advantage of not fluctuating across time. Finally, we limited this study to the testing of the stoichiometric imbalance hypothesis without identifying specific pathological mechanisms which remains the ultimate goal of molecular genetic research in BD.

### Conclusion

We developed a method for measuring SI in the expression of BD GWAS genes. We found that many genes displayed either a relative over- or under-expression in cases, and that these patterns were similar across datasets. We used these gene-level measures of SI to compute the predicted probability of BD and found that the fraction of phenotypic variation explained by this probability is many times higher than what is achieved by using absolute expression values or any PRS measure. The strength of these results suggests that dysregulation of stoichiometric balance is an important factor in BD aetiology and raises the question of whether it may also be central to other pathologies.

### Supplementary information

Supplemental figures

Supplemental tables

## Data Availability

All data used in this study are available through the PsychENCODE Consortium. Access to the data is managed by the NIMH Repository and Genomics Resource, and the data are distributed via Synapse under the CommonMind HBCC (syn10623034), CommonMind CMC-Pitt (syn8241760), BrainGVEX (syn3270015) and BipSeq (syn5844980) studies.

## Code availability

Scripts and code used for the analysis in this manuscript are available at https://github.com/comgen/SI_RNAseq.
