# Supplementary material for: Bipolar patients display stoichiometric imbalance of gene expression in post-mortem brain samples
Source: Mol Psychiatry. 2024 Feb 13;29(4):1128–38. doi: 10.1038/s41380-023-02398-0 (PMC11176081; doi:10.1038/s41380-023-02398-0)
Supplement: Supplementary file 1 — Supplemental figures [file 41380_2023_2398_MOESM1_ESM.docx]

**Supplementary materials**

Complete acknowledgement text for all datasets

**CommonMind Consortium**

Data were generated as part of the CommonMind Consortium supported by funding from Takeda Pharmaceuticals Company Limited, F. Hoffman-La Roche Ltd and NIH grants R01MH085542, R01MH093725, P50MH066392, P50MH080405, R01MH097276, RO1-MH-075916, P50M096891, P50MH084053S1, R37MH057881, AG02219, AG05138, MH06692, R01MH110921, R01MH109677, R01MH109897, U01MH103392, and contract HHSN271201300031C through IRP NIMH. Brain tissue for the study was obtained from the following brain bank collections: the Mount Sinai NIH Brain and Tissue Repository, the University of Pennsylvania Alzheimer’s Disease Core Center, the University of Pittsburgh NeuroBioBank and Brain and Tissue Repositories, and the NIMH Human Brain Collection Core. CMC Leadership: Panos Roussos, Joseph Buxbaum, Andrew Chess, Schahram Akbarian, Vahram Haroutunian (Icahn School of Medicine at Mount Sinai), Bernie Devlin, David Lewis (University of Pittsburgh), Raquel Gur, Chang-Gyu Hahn (University of Pennsylvania), Enrico Domenici (University of Trento), Mette A. Peters, Solveig Sieberts (Sage Bionetworks), Thomas Lehner, Stefano Marenco, Barbara K. Lipska (NIMH).

**PsychEncode Consortium**

Data were generated as part of the PsychENCODE Consortium, supported by: U01DA048279, U01MH103339, U01MH103340, U01MH103346, U01MH103365, U01MH103392, U01MH116438, U01MH116441, U01MH116442, U01MH116488, U01MH116489, U01MH116492, U01MH122590, U01MH122591, U01MH122592, U01MH122849, U01MH122678, U01MH122681, U01MH116487, U01MH122509, R01MH094714, R01MH105472, R01MH105898, R01MH109677, R01MH109715, R01MH110905, R01MH110920, R01MH110921, R01MH110926, R01MH110927, R01MH110928, R01MH111721, R01MH117291, R01MH117292, R01MH117293, R21MH102791, R21MH103877, R21MH105853, R21MH105881, R21MH109956, R56MH114899, R56MH114901, R56MH114911, R01MH125516, and P50MH106934 awarded to: Alexej Abyzov, Nadav Ahituv, Schahram Akbarian, Alexander Arguello, Lora Bingaman, Kristin Brennand, Andrew Chess, Gregory Cooper, Gregory Crawford, Stella Dracheva, Peggy Farnham, Mark Gerstein, Daniel Geschwind, Fernando Goes, Vahram Haroutunian, Thomas M. Hyde, Andrew Jaffe, Peng Jin, Manolis Kellis, Joel Kleinman, James A. Knowles, Arnold Kriegstein, Chunyu Liu, Keri Martinowich, Eran Mukamel, Richard Myers, Charles Nemeroff, Mette Peters, Dalila Pinto, Katherine Pollard, Kerry Ressler, Panos Roussos, Stephan Sanders, Nenad Sestan, Pamela Sklar, Nick Sokol, Matthew State, Jason Stein, Patrick Sullivan, Flora Vaccarino, Stephen Warren, Daniel Weinberger, Sherman Weissman, Zhiping Weng, Kevin White, A. Jeremy Willsey, Hyejung Won, and Peter Zandi.

**
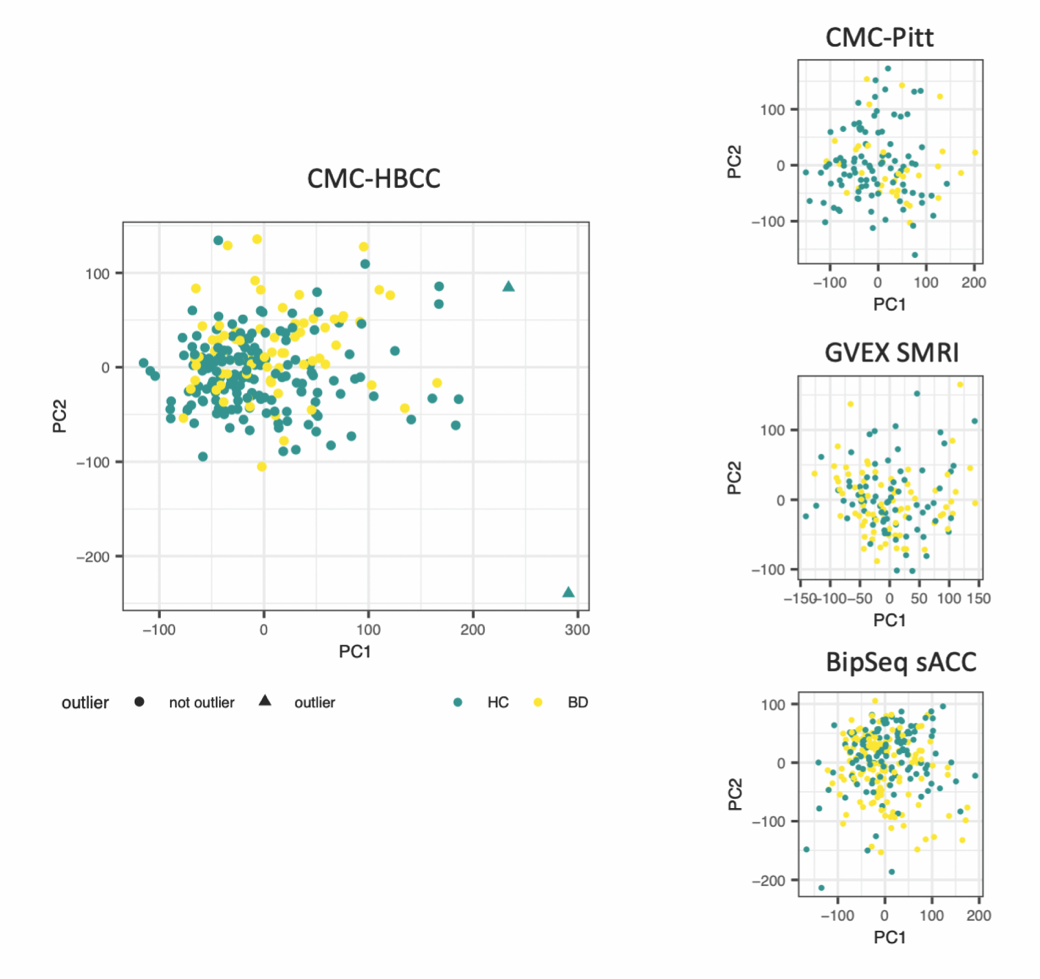
**

**Figure S1 - PCA for the RNAseq datasets**

Principle component analysis of the RNAseq expression data (log2 RPKM). Samples deemed outliers depicted with triangles. Controls (green), and cases (yellow).

**
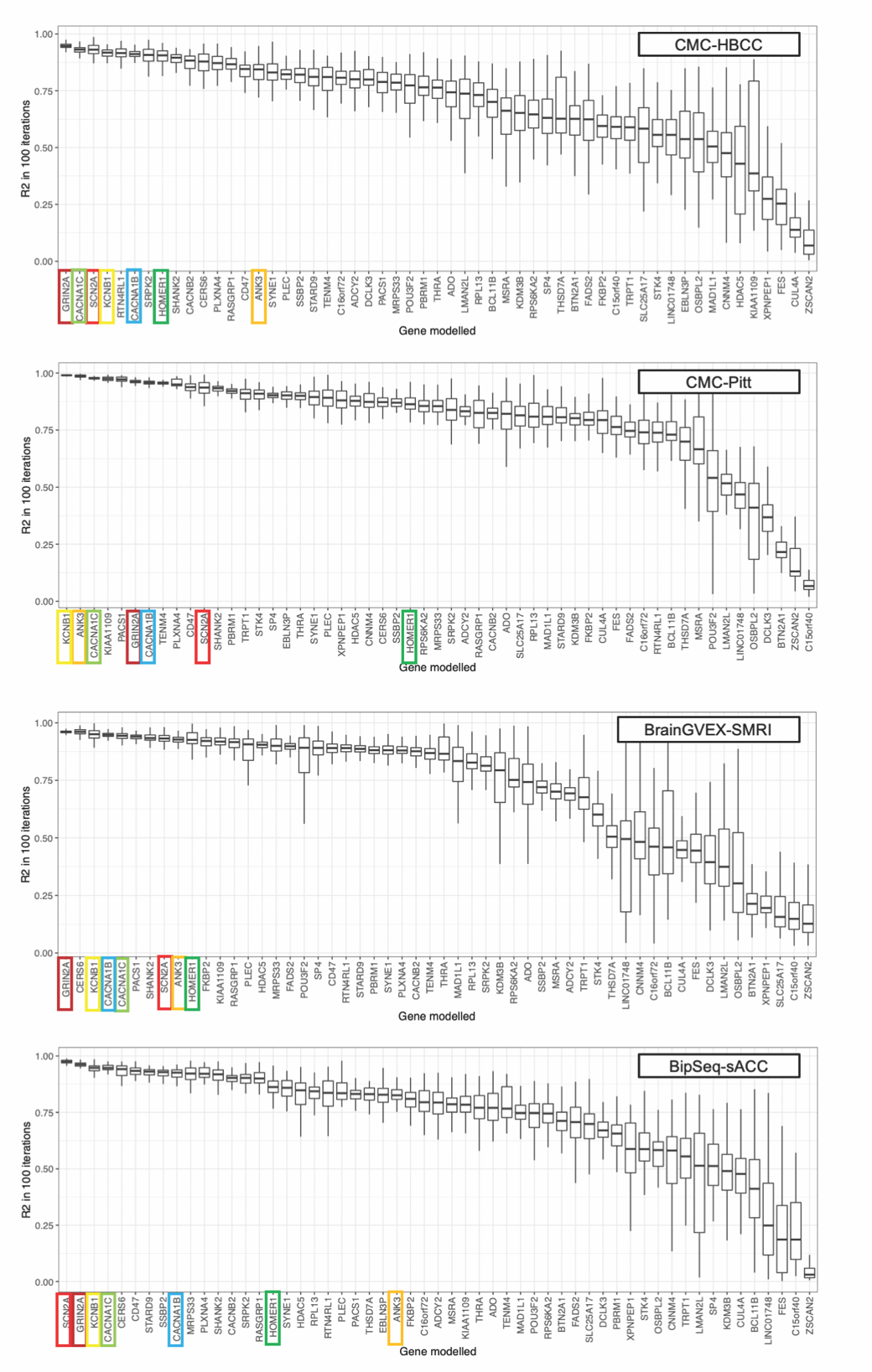
**

**Figure S2 – R^2^ distribution across iterations for each modelled gene**

Genes ordered according to decreasing median R^2^ of the model. Colour highlighting of a selection of genes that place consistently amongst the models with best fit.


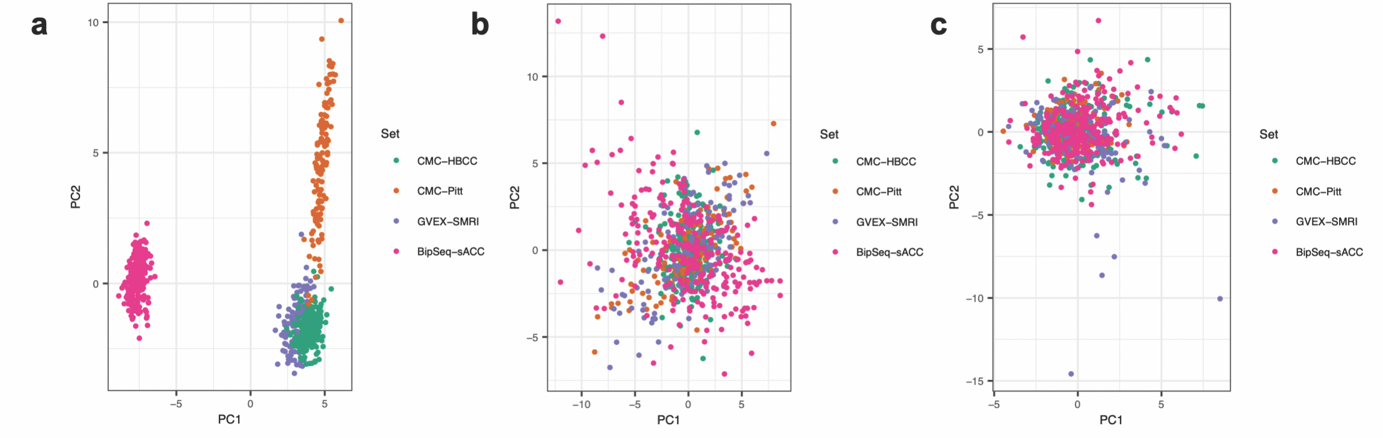


**Figure S3 - PCA for gene expression and sWSR**

1. Principle component analysis of the RNAseq expression data of the genes of interest (log2 RPKM).
2. Principle component analysis of the RNAseq expression data of the genes of interest (log2 RPKM), residualized for covariates.
3. Principle component analysis of the sWSR, comparing all four datasets.


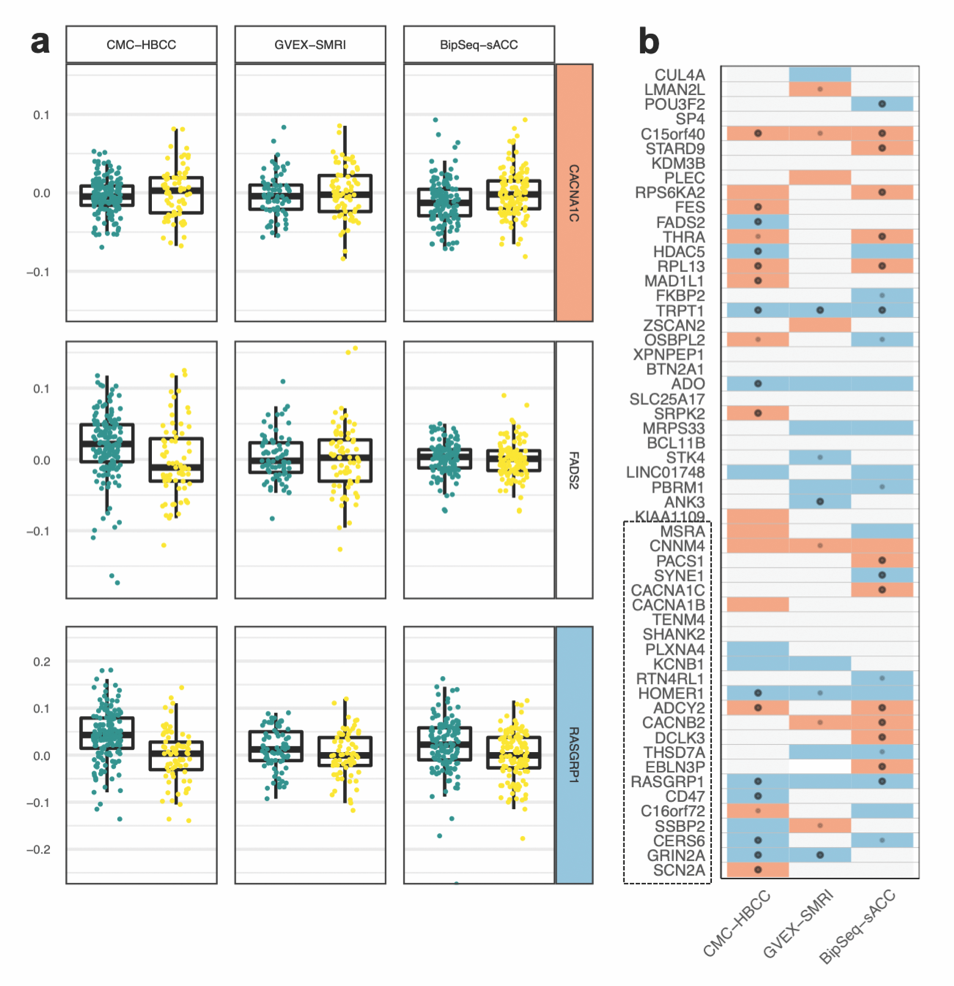


**Figure S4– Patterns of case-control differences in WSR when modelling with BD samples**

**a.** HC and BD residuals (${WSR}_{i,j}$) for three example genes in the three datasets with a sufficient number of BD samples. For *CACNA1C* (top) cases have higher observed expression than predicted. For *FADS2* (middle), there is no pattern of difference between controls and cases, and for *RASGRP1* (bottom) cases have lower observed expression than predicted. (A total of 12 points are outliers not shown in the plot).

**b.** P-values from the comparison between HC and BD mean residuals for all PGC3 associated genes for all three datasets (gene ordering identical to Figure 2, dotted line delineates boundary of large module of co-expression). Colours indicate direction of difference; red higher in BD and blue lower in BD (p < 0.15). Small dots indicate nominal significance (p < 0.05) and large dots indicate significant p-value (FDR < 0.05 across all genes and datasets).
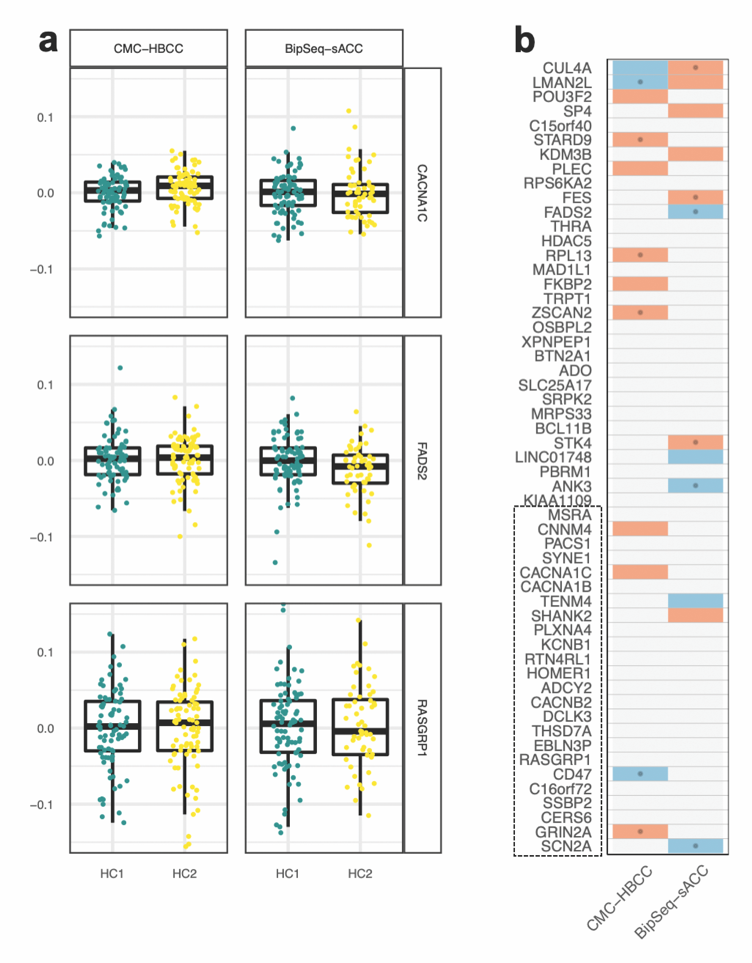


**Figure S5 – Patterns of HC1-HC2 differences in WSR when modelling with HC1 samples:** c**ontrol samples from each dataset are divided into two sets: HC1 is used for modelling with random resampling, whilst HC2 samples are not used for modelling.**

**a.** HC1 and HC2 residuals (${WSR}_{i,j}$) for three example genes in the two datasets with a large number of controls. For *CACNA1C* (top), *FADS2* (middle), and *RASGRP1* (bottom) there is no pattern of difference between controls and cases.

**b.** P-values from the comparison between HC1 and HC2 mean residuals for all PGC3 associated genes for two datasets (modelling with a subset of HC samples was not possible for CMC-Pitt or BrainGVEX-SMRI due to their limited HC sample size.) Gene ordering identical to Figure 2, dotted line delineates boundary of large module of co-expression. Colours indicate direction of difference; red higher in BD and blue lower in BD (p < 0.15). Small dots indicate nominal significance (p < 0.05) and large dots indicate significant p-value (FDR < 0.05 across all genes and datasets).


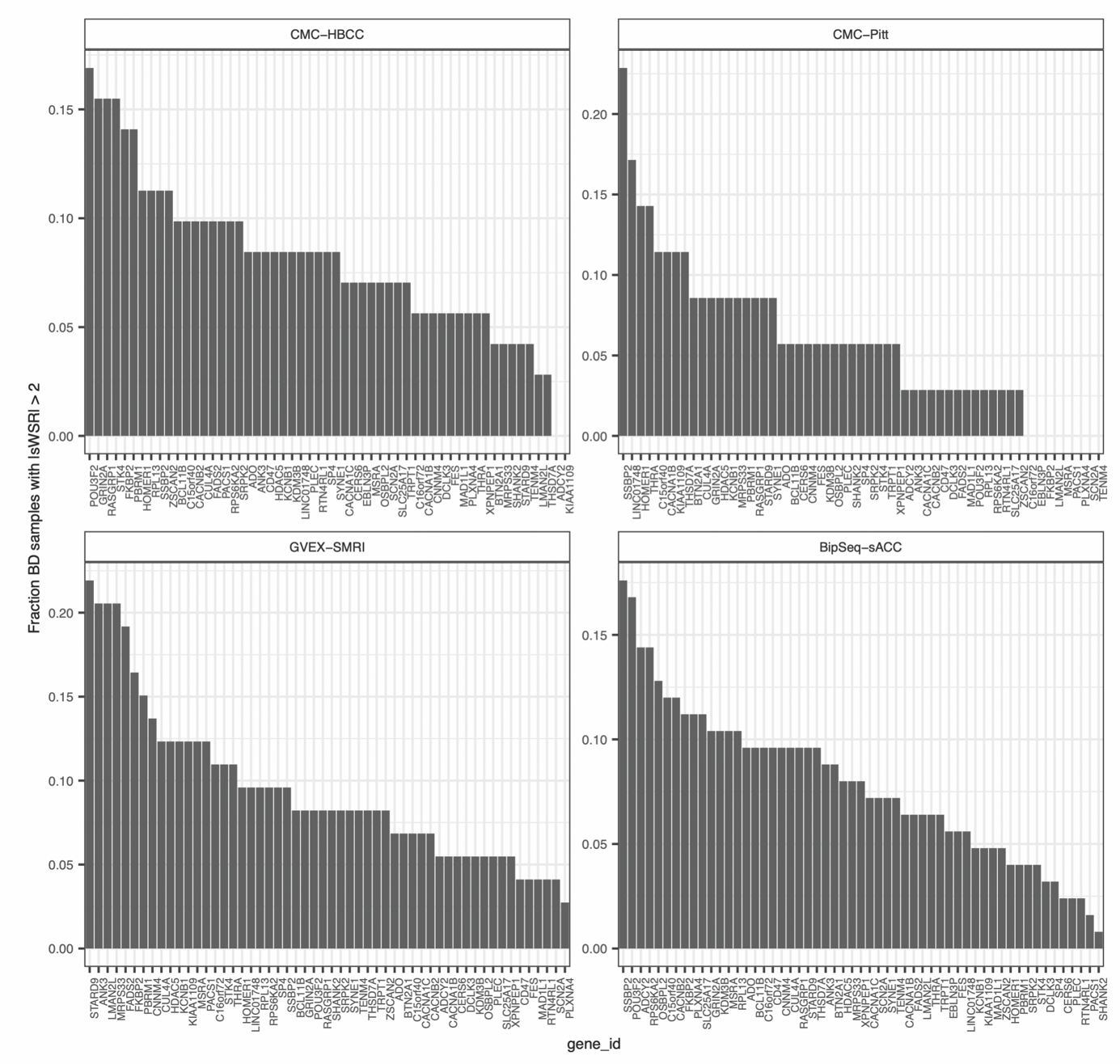


**Figure S6: Proportion of BD samples in which genes are stoichiometrically imbalanced.**

Stoichiometrically imbalanced is defined as $\left| {sWSR}_{i,j} \right|>2$.
